# From body to mind: how body-mind axial awareness training enhances body awareness, mindfulness, positive embodiment and mental health in healthcare students

**DOI:** 10.1080/21642850.2026.2656029

**Published:** 2026-04-10

**Authors:** Yi-Min Tien, Chia-Yao Lin, Yunn-Wen Lien, Li-Chuan Hsu

**Affiliations:** a Department of Psychology, Chung Shan Medical University, Taichung, Taiwan; b Clinical Psychological Room, Chung Shan Medical University Hospital, Taichung, Taiwan; c School of Medicine, China Medical University, Taichung, Taiwan; d Department of Psychology, National Taiwan University, Taipei, Taiwan; e Graduate Institute of Biomedical Sciences, China Medical University, Taichung, Taiwan

**Keywords:** Body-Mind Axial Awareness (BMAA), body awareness, mindfulness, positive embodiment, mental health

## Abstract

**Background:**

Healthcare students face intense emotional and academic stress, leading to anxiety and poor mental health. Body-Mind Axial Awareness (BMAA)—a movement-based, body-centered practice—integrates bodily awareness and interoceptive attention to promote mind–body regulation and mental health.

**Methods:**

A pretest–posttest controlled design was used with 38 students in the BMAA group and 27 in the control group. Group differences arising from non-random enrollment were statistically adjusted using analysis of covariance (ANCOVA), and within-group changes were examined using paired t-tests and repeated-measures multivariate analysis of variance (MANOVA). Participants completed eight weeks of BMAA training involving weekly two-hour sessions and daily home practice. Assessments before and after intervention included four domains of psychological functioning, measured using validated instruments: body awareness (Body Awareness Ability Inventory; Multidimensional Assessment of Interoceptive Awareness), mindfulness (Mindful Attention Awareness Scale), positive embodiment (Experience of Embodiment Scale), and mental health (Adult Mental Health Scale; Toronto Alexithymia Scale-20; State–Trait Anxiety Inventory-Trait; Satisfaction with Life Scale).

**Results:**

After controlling for baseline differences, the BMAA group showed greater gains than the control group in body awareness, mindfulness, positive embodiment, and mental health. Within-group analyses further indicated that BMAA participants demonstrated significant pre–post improvements across most body awareness and mindfulness subscales (except Not-Distracting and Not-Worrying), as well as across embodiment dimensions (except Resisting Objectification), along with enhanced emotional awareness and expression, reduced anxiety, and higher life satisfaction (*p* < .05 for relevant comparisons). Despite the absence of random assignment and modest group-size imbalance, ANCOVA and within-group findings demonstrated consistent effects.

**Conclusion:**

The findings demonstrate that BMAA training enhances multiple facets of body–mind functioning, including bodily awareness, mindfulness, positive embodiment, and mental health. As healthcare students encounter cognitive and emotional demands, BMAA may offer a feasible embodied approach for strengthening psychological resilience and adaptive stress management during professional training.

## Introduction

In today’s digital age, university students are constantly bombarded with information, directing much of their attention toward external stimuli and societal expectations while leaving little room for introspection or self-care. In turn, this external focus of attention has contributed to a significant rise in anxiety and emotional distress among students (Bayram & Bilgel, 2008; Regehr et al., 2013; Storrie et al., 2010). In the United States, up to 81.7% and 85.6% of university students report experiencing situational anxiety and trait anxiety, respectively (Brenneisen Mayer et al., 2016). Longitudinal studies indicate that student anxiety disorders have doubled since 2008 (Scheffler et al., 2019), with the COVID-19 pandemic further exacerbating these issues (Mari & Oquendo, 2020).

Similar patterns have been observed in populations in Taiwan, where the present study was conducted. Epidemiological data show a steady increase in anxiety and common mental disorders over the past decade, with recent surveys reporting elevated anxiety levels across both youth and adult groups (Ministry of Health & Welfare, 2024; Fu et al., 2013; Wong et al., 2020). These trends underscore the growing mental health burden among university students in this cultural context and highlight the importance of effective preventive and intervention strategies.

Healthcare students—across medicine, nursing, pharmacy, dentistry, physical therapy, and public health programmes—show elevated levels of trait anxiety and psychological distress (Dyrbye et al., 2005; Rotenstein et al., 2016; Tian-Ci Quek et al., 2019; Tung et al., 2018). These mental health challenges have intensified since the COVID-19 pandemic, with meta-analyses reporting that over one-third of healthcare students experience clinically significant anxiety or depression, exceeding rates observed in general university populations (Lasheras et al., 2020). Persistent depression and anxiety can disrupt the integration between cognitive–affective processes and physiological states—i.e. mind–body disconnection—leading to distorted perceptions of bodily sensations and maladaptive feedback loops between arousal and cognitive interpretation (Mallorquí-Bagué et al., 2016). This mind–body disconnection negatively impacts academic performance (Chandavarkar et al., 2007), self-esteem (Sowislo & Orth, 2013), and stress regulation (Wang et al., 2016), and may further contribute to impaired concentration, social withdrawal, technology addiction (Small et al., 2020), procrastination (Custer, 2018), and sleep disturbances. Over time, this dysregulated mind-body integration can manifest as psychosomatic symptoms, including palpitations, gastrointestinal discomfort, and muscle tension (Eller et al., 2006; Mallorquí-Bagué et al., 2016), which are mediated by heightened autonomic arousal and misinterpreted interoceptive signals (Critchley & Harrison, 2013; Domschke et al., 2010; Paulus & Stein, 2006). In severe cases, persistent mind-body disconnection and psychosomatic symptomatology may increase the risk of self-harm and suicide (Blacker et al., 2019).

Given these widespread disruptions in mind–body integration, recent research has increasingly emphasised the need for interventions that directly target bodily awareness and emotional regulation. Although traditional psychological therapies primarily focus on modifying maladaptive thoughts, such approaches may be limited in addressing embodied aspects of emotional distress (Blagys & Hilsenroth, 2002; Kannan & Levitt, 2013). In contrast, body-centred practices—such as Qigong, body therapy, meditation, and yoga—have gained attention as alternative approaches (Gyllensten et al., 2003; Lipnicki & Byrne, 2008; Neumark-Sztainer et al., 2018). These practices emphasise bodily awareness and mind–body connection, offering a more integrative pathway to emotional regulation than interventions targeting cognition alone (Klatte et al., 2016; Strauss et al., 2014).

Gyllensten et al. (2010) proposed that body awareness is fundamental to “making the body an agent of experience,” enabling individuals to develop a deeper connection with themselves through inner perception. This heightened awareness allows individuals to recognise bodily sensations and internal cues, shifting the body from a passive entity to an active participant in shaping perception and responses to the world. By becoming more attuned to one’s physical and emotional signals—such as muscle tension, breath patterns, or an increased heart rate—individuals are able to regulate their emotions through techniques like breath control or movement adjustments (Arch & Craske, 2006; Goldin & Gross, 2010; Gyllensten et al., 2003). Cultivating body awareness facilitates the perception and release of these stored emotions, fostering self-awareness and emotional regulation (2009; Gyllensten et al., 2003; Malmgren-Olsson et al., 2001). Therefore, these findings highlight the importance of integrating body awareness into therapy as a key component in promoting emotional and psychological well-being (Davis, 2021; Rosendahl et al., 2021).

The present study approaches mental health challenges through the “embodied mind” framework, which highlights the interconnectedness of the body, mind, and environment (Rosendahl et al., 2021; Varela et al., 2017). This perspective acknowledges that cognition and emotions are not merely mental processes but are deeply rooted in bodily sensations and lived experiences. Therefore, we introduce the concept of “embodiment” to emphasise individuals’ subjective experiences of living in the body, encouraging a shift from externally imposed standards and social expectations to a more internally grounded and integrated self-understanding (Piran & Teall, 2012; Piran, 2002).

An individual’s psychological and physical well-being is shaped by past bodily experiences (Piran, 2017). To systematically assess these experiences, Piran (2020) developed the Experience of Embodiment Scale, which conceptualises embodiment as a reflection of the mind-body relationship. Positive embodiment arises when individuals respect and engage harmoniously with their bodies, prioritising overall well-being rather than adhering to idealised beauty standards (Piran, 2020). This state is marked by intuitive self-care practices, such as eating in response to hunger, resting when fatigued, and participating in enjoyable physical activities (Tien et al., 2025). Such engagement fosters self-efficacy, self-agency, and empowerment, ultimately contributing to lower stress, anxiety, and depression (Alleva et al., 2015; Piran, 2017; Tiggemann & Zaccardo, 2015). In contrast, negative embodiment reflects a disconnection from the body, leading to the neglect of self-care as well as increased stress, anxiety, and emotional distress (Piran, 2020). Recognising this contrast in experiences of embodiment and its effects on mental health underscores the need for interventions that foster a positive mind-body relationship, further reinforcing the significance of body awareness in promoting overall well-being.

### Understanding body-mind axial awareness (BMAA)

BMAA is a culturally grounded embodied practice developed to enhance psychological and physical well-being. Originating from ***Ya-Yue***—an ancient Chinese ceremonial dance associated with heavenly worship—BMAA draws on Confucian ideals of self-cultivation and moral development (Chen, 2011a, Chen, 2011b). Building on these traditional foundations, researchers at National Taiwan University transformed BMAA into a structured training programme aimed at fostering self-awareness, self-regulation, and personal agency (Lien et al., 2019; Teng & Lien, 2016). Central to BMAA is an emphasis on embodied regulation and cultivated tranquility—an approach grounded in classical Confucian traditions of self-cultivation and moral refinement (Yang, 1996).

At the core of BMAA is the principle of maintaining an imagined vertical axis—from the top of the head to the perineum—which serves as both a physical and mental anchor (Lien et al., 2019; Teng & Lien, 2016). Unlike practices such as Tai Chi, which involve memorising complex sequences, BMAA emphasises simple, isolated movements that allow for intuitive engagement with the axial concept. Beginners often start with lying-down exercises to promote relaxation and bodily awareness before progressing to more dynamic movements. Through focused engagement with the body, practitioners learn to recognise patterns of muscular tension and release, understand how the body coordinates with the central axis, and improve both bodily awareness and movement efficiency.

Existing studies indicate that BMAA exerts multidimensional benefits on both psychological and physical health. Among university students, Teng & Lien, (2016) found that a 12-hour BMAA programme significantly enhanced executive functions, particularly working memory, compared with Chan meditation and waitlist controls. In children, Yeh et al., (2023) reported that a 6-day BMAA camp improved sustained attention and body balance. Among elementary school teachers, Weng, (2020) showed that a 6-week BMAA intervention increased interoceptive awareness, resilience, and sleep quality while reducing perceived stress and negative affect. Additionally, Hsu, (2020) demonstrated in a randomised controlled trial that a 2-week short-form BMAA programme reduced depressive symptoms, improved emotion regulation, and enhanced body awareness and working memory in university students. Beyond intervention studies, Lien et al., (2019) provided narrative and empirical evidence that BMAA and its origins in Ya-Yue promote creativity, motor coordination, sustained attention, and reduce mind-wandering (See Supplement).

### BMAA practice

BMAA was developed by researchers at the Taiwan Body-Mind Axial Awareness Centre (https://bmaa.tw/) and integrates dynamic axial-alignment movements with deep self-massage techniques. The core principle involves directing relaxation during exhalation toward specific support points and their corresponding resistance surfaces, allowing targeted body regions to gradually sink, soften fascial tension, and release muscular knots while maintaining relaxation in other areas. As described by Chen (2011b), this coordinated practice helping loosen rigid musculature and fascia, fostering a sense of embodied wholeness, and providing broad somatic and mind–body therapeutic benefits (Hsu, 2020; Lien et al., 2019; Teng & Lien, 2016; Weng, 2020; Wu et al., 2023).

BMAA is typically conducted in small-group classes (6–12 participants) or through one-on-one in-person instruction to ensure individualised guidance and posture correction. All sessions are led by experienced instructors who have practiced BMAA for several years and completed formal certification and mentorship training through the Taiwan BMAA Centre. Each standard session lasts approximately 120 minutes, during which participants engage in movements at their own pace while adhering to Ya-Yue principles and incorporating relaxation techniques such as chest–abdominal breathing and full-body fascial release using tools like tennis balls. Classes take place in spaces equipped with floor mats, allowing participants to lie down comfortably in a warm and quiet environment—an essential condition for facilitating deep relaxation and body awareness.

The practice emphasises activation and sensing of the fascia system, proprioceptive and interoceptive organs, and key structural regions, including the upper and lower limbs, scapulae, pelvic floor, and spinal axis. Beginners typically start with lying-down exercises to enhance sensory awareness and relaxation before progressing to more dynamic axial movements.

### Main purpose

Grounded in the embodied mind framework, this study shifts the focus from traditional cognition-based mental health interventions (Blagys & Hilsenroth, 2002; Kannan & Levitt, 2013; Sahranavard et al., 2018) to body-centred practices that cultivate bodily awareness and lived experience. BMAA, a movement-based practice, aims to enhance self-awareness, embodied experience, and mind–body integration. Previous studies have shown that BMAA benefits multiple domains of health. However, despite these promising findings, most studies are limited by small sample sizes, lack of control groups, or focus on non-clinical adult or child populations. To date, no study has systematically examined the effects of BMAA on healthcare professional students—a population at heightened risk of anxiety, stress, and mind–body disconnection, especially in the post-pandemic context.

To address this gap, the present study employed a pretest–posttest control group design and administered eight validated questionnaires across four domains—body awareness, mindfulness awareness, embodiment experience, and mental health—to evaluate the effects of BMAA training. Specifically, we aim to determine whether BMAA can (1) reduce anxiety and psychological distress, and (2) enhance body awareness, positive embodiment, and overall well-being by strengthening mind–body integration. This study provides empirical evidence for embodied interventions in healthcare education and offers a practical model for integrating body-based practices into mental health promotion.

## Methods

We recruited 38 undergraduate students (30 females; mean age = 21.61, SD = 3.07) from China Medical University as the experimental group. All participants were second-year students or above and voluntarily enroled in the “Brain and Emotions” elective course, which incorporated the eight-week BMAA programme. The sample included 22 medical, 10 pharmacy, 3 dental, 2 traditional Chinese medicine, and 1 public health student. A control group of 27 medical students (14 females; mean age = 18.89, SD = 1.91) in their first or second academic year was recruited through convenience sampling from the same university. These students were not enroled in the elective course. The age difference between groups was significant (*t*(63) = 4.07, *p* < .001).

Participants in the experimental group were recruited and completed the intervention first, followed by the recruitment of the control group. All participants reported no medication use and no prior experience with meditation or yoga. Ethical approval was obtained from the Ethics Review Committee (CRREC-112-078), and informed consent was collected from all participants.

### Study design and procedure

This study was conducted as part of the 18-week elective course “Brain and Emotions.” It adopted a non-randomised pretest–posttest control group design. Students who voluntarily enroled in the course formed the experimental group, while the control group consisted of medical students who did not participate in the intervention. The experimental group received an eight-week BMAA training programme during Weeks 6–13 of the semester. Participants attended one two-hour session per week, accompanied by daily home practice and weekly written reflections. The control group did not receive any intervention.

All BMAA sessions were conducted in the university’s dance studio—a spacious venue with a wooden floor and interlocking EVA mats that provided a comfortable and safe environment for movement practice. Each participant was provided with two EVA exercise mats (96 × 96 × 2 cm) combined as a personal practice area. The training was delivered in small-group sessions to maintain close instructor–student interaction.

The sessions were led by a primary instructor with over ten years of BMAA learning and practice experience, who has consistently offered at least one BMAA course every semester for the past three years. The instructor also received weekly professional supervision from the Taiwan Body-Mind Axial Awareness Centre. Two trained teaching assistants supported each class by providing posture correction, safety assistance, and individualised feedback.

Each two-hour session followed a consistent structure consisting of guided movement practice, group discussion, and optional individual consultation. Students who had questions about movement techniques, bodily sensations, or the application of BMAA principles in daily life could remain after class for personalised guidance. Sessions concluded with brief sharing and reflection activities to consolidate learning and deepen awareness.

**Figure 1.**
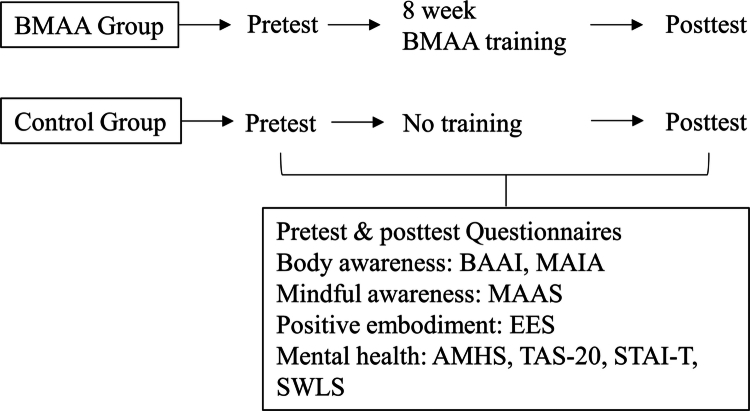
Study design and assessment procedure. Flow diagram of the study design. The BMAA group received eight weeks of training between pretest and posttest, while the control group received no intervention. Both groups completed pretest and posttest questionnaires assessing body awareness (Body Awareness Ability Inventory, BAAI; Multidimensional Assessment of Interoceptive Awareness, MAIA), mindful awareness (Mindful Attention Awareness Scale, MAAS), positive embodiment (Experience of Embodiment Scale, EES), and mental health (Adult Mental Health Scale, AMHS; Toronto Alexithymia Scale-20, TAS-20; State–Trait Anxiety Inventory–Trait, STAI-T; Satisfaction with Life Scale, SWLS).

Participants completed a pretest before the intervention and a posttest after the final session. The same set of questionnaires—assessing body awareness, mindfulness, positive embodiment, and mental health—was administered one week before and one week after the training. Each assessment took approximately 45 minutes to complete. The control group completed identical pretest and posttest measures but did not receive any intervention or home practice assignments (see Figure 1).

## Questionnaires

The primary research tools used in this study were self-report questionnaires. Below is a detailed list of the scales we utilised.

### Body awareness

#### Body awareness ability inventory(BAAI)

The BAAI (Tsao, 2007) assesses body awareness across five subdomains—Body Usage, Muscle Tension, Body Control, Bodily Abnormalities, and Breathing—using 21 items rated on a Likert scale. It has good internal consistency (Cronbach’s *α* = .78– .82; total *α* = .89) and sensitivity to change following mind–body interventions. For instance, Chang et al. (2014) reported improved BAAI scores after group-based training among adolescents with pronounced somatic symptoms. In the present study, the BAAI demonstrated excellent internal consistency (Cronbach’s *α* = .89), indicating strong reliability within our sample.

#### Multidimensional assessment of interoceptive awareness (MAIA)

The MAIA (Mehling et al., 2012) is a 32-item measure comprising eight dimensions of interoceptive awareness: Noticing, Not-Distracting, Not-Worrying, Attention Regulation, Emotional Awareness, Self-Regulation, Body Listening, and Trusting. Previous validation research has shown acceptable to strong reliability across subscales (Cronbach’s *α* = .66–.82), and the Chinese version similarly demonstrates strong psychometric properties (overall *α* = .91; Lin et al., 2017).

In this study, internal consistency for the MAIA subscales was as follows: Noticing (*α* = .71), Not-Distracting (*α* = .23), Not-Worrying (*α* = .71), Attention Regulation (*α* = .77), Emotional Awareness (*α* = .73), Self-Regulation (*α* = .86), Body Listening (*α* = .80), and Trusting (*α* = .88). These values generally fall within the acceptable-to-strong range, with the exception of Not-Distracting (*α* = .23). Although only the Not-Distracting subscale showed low internal consistency in our sample, lower reliabilities for both the Not-Distracting and Not-Worrying subscales have been reported in the original MAIA as well as in several cross-cultural validation studies, including the Chinese version. This pattern suggests that these brief, reverse-scored subscales are psychometrically weaker and may be particularly sensitive to cultural differences in coping with bodily discomfort (Ferentzi et al., 2021; Mehling et al., 2012).

### Mindful awareness

#### Mindful awareness attention scale (MAAS)

The MAAS (Brown & Ryan, 2003) is a 15-item unidimensional measure of present-moment awareness. It has shown strong psychometric properties, including a four-week test–retest reliability of *r* = .81 in the original validation (Brown & Ryan, 2003). Its temporal stability has also been supported in subsequent studies, which reported intraclass correlation coefficients (ICCs) ranging from .80 to .85 across multiple samples (Black et al., 2012; MacKillop & Anderson, 2007). Internal consistency is consistently high, with Cronbach’s *α* typically falling between .82 and .87 (Feldman et al., 2007). In the present study, the MAAS demonstrated similarly strong reliability (Cronbach’s *α* = .86).

### Positive embodiment

#### Experience of embodiment scale (EES)

The EES (Piran, 2020) assesses six dimensions of embodiment—Agency and Functionality (AF), Experience and Expression of Sexual Desire (EESD), Attuned Self-Care (ASC), Body Unencumbered Adjustment (BUA), Positive Body Connection and Comfort (PBCC), and Resisting Objectification (RO). Prior research demonstrates excellent reliability for the total scale (*α* = .94) and strong psychometric performance of the Chinese version (*α* = .90; Tien et al., 2025). In the current study, the EES continued to show high internal consistency (Cronbach’s *α* = .88), supporting its suitability for assessing positive embodiment among Taiwanese healthcare students.

### Mental health

#### Adult mental health scale (AMHS)

The AMHS (Huang et al., 2011) assesses mental health across five domains: Somatic Symptoms, Anxiety, Social Undermining, Depression, and Positive Mentality. It demonstrates high reliability (*α* = .92) and good convergent validity, with negative correlations with the Beck Depression Inventory-II (r = −.38 to −.64). These findings support the AMHS as a reliable and valid measure of adult mental health. Consistent with previous findings, the AMHS demonstrated excellent reliability in this study (Cronbach’s *α* = .93).

#### Toronto alexithymia scale-20 (TAS-20)

The TAS-20 (Bagby et al., 1994) is a 20-item measure assessing alexithymia across three dimensions: Difficulty Identifying Feelings, Difficulty Describing Feelings, and Externally Oriented Thinking. It has demonstrated good internal consistency in prior research (total *α* ≈ .81; Leising et al., 2009), with similar reliability reported in the Taiwanese version (Lin & Chan, 2003). In the present study, the TAS-20 showed acceptable internal consistency (Cronbach’s *α* = .77), consistent with established norms.

#### State-trait anxiety inventory (STAI-T)

The STAI (Spielberger, 1983) is a 40-item self-report measure consisting of two 20-item subscales assessing state and trait anxiety. This study used only the Trait Anxiety (STAI-T) subscale, which assesses individuals’ general disposition toward anxiety. The Chinese version was translated and validated by Chung & Long, (1984). The STAI-T has shown high internal consistency (*α* ≈ .86–.95) and acceptable test–retest reliability (*r* ≈ .65–.75 over two months) across diverse adult populations (Barnes et al., 2002; Spielberger, 1983). A Danish validation study further reported excellent reliability (Cronbach’s *α* = .93; Intraclass coefficient = .80) (Gustafson et al., 2020).

The Chinese version demonstrates robust reliability (Cronbach’s *α* = .92 and a 2-week test–retest *r* = .91) among Taiwanese outpatients (Ma et al., 2012), and consistent factorial validity among Chinese university students (Han et al., 2020). Early validation studies also confirmed high internal consistency in Chinese adolescent samples (Shek, 1993, 1988). In the present study, it showed excellent internal consistency (Cronbach’s *α* = .92), consistent with these established norms.

#### Satisfaction with life scale (SWLS)

The SWLS (Diener et al., 1985) is a five-item measure assessing overall life satisfaction. The Chinese version (Wu & Yao, 2006) has demonstrated strong internal consistency (Cronbach’s *α* = .89) and factorial stability across genders. The scale also shows good criterion-related validity, correlating positively with positive affect and interpersonal relationships and negatively with negative affect and neuroticism. In this study, it demonstrated strong reliability (Cronbach’s *α* = .87).

### Statistical analysis

All statistical analyses were conducted using SPSS (Version 28). Assumptions of normality and homogeneity of variance were tested and met for all dependent variables prior to hypothesis testing. A priori power analysis for Analyses of covariance (ANCOVAs) (*α* = .05, power = .80, medium effect *f* = .25) indicated a minimum required sample of 52 participants (Faul et al., 2007), and our final sample of 65 exceeded this threshold, supporting adequate statistical power for the planned analyses.

To examine the effects of the BMAA intervention, a two-step analytical strategy was adopted:

(1) Between-group comparison (Experimental vs. Control):

ANCOVAs were performed with posttest scores as dependent variables, group (experimental vs. control) as the between-subjects factor, and pretest scores entered as covariates. This approach was chosen because the experimental group was not randomly assigned and may have differed from the control group at baseline. By statistically adjusting for these potential pre-existing disparities, ANCOVA yields a more accurate estimation of the intervention effect than repeated-measures ANOVA, which presumes initial group equivalence and does not account for such differences.

(2) Within-group change in the experimental group

To further examine whether participants in the experimental group showed significant improvements following the BMAA intervention, two complementary analyses were conducted.

2-1. Paired-samples t-tests:

For the psychological measures that consisted of a single total score (i.e. representing one dependent variable), paired-samples t-tests were conducted to evaluate pre–post differences within the experimental group.

2-2. Repeated-measures MANOVA:

Two multidimensional questionnaires—the MAIA and EES—were analysed using repeated-measures MANOVA. Both instruments consist of multiple subscales that may be intercorrelated; therefore, MANOVA was selected to account for potential interaction or covariance among subscales and to assess overall multivariate changes from pre- to post-intervention.

For all analyses, effect sizes (*η*² for ANCOVA and partial *η*² for MANOVA) were reported for significant and marginal effects. Post hoc power analyses were performed for results approaching significance to ensure sufficient statistical sensitivity. The alpha level was set at *p* < .05.

## Results

Prior to training, no significant differences were found between the BMAA training group and the control group on most dependent variables, except for the ASC and RO subscales of the EES. The training group had higher ASC and RO scores than the control group in the pretest (both *t* > 3.89, *p* < .01 for relevant comparisons). To account for these pretest differences, ANCOVAs were conducted.

### Body awareness and mindfulness

**Between-group comparison.** ANCOVA results for BAAI showed that the experimental group had significantly higher posttest scores than the control group after controlling for pretest differences (*F*(1, 62) = 7.45, *p* < .01, *η*² = .11, power = 0.77). Similarly, MAIA analysis indicated that the BMAA group demonstrated significantly higher adjusted post-intervention scores across several dimensions, including ‘Noticing’ (*F*(1, 62) = 5.35, *p* < .05, *η²* = .08, power = 0.63), ‘Attention Regulation’ (*F*(1, 62) = 4.70, *p* < .05, *η²* = .07, power = 0.57), ‘Emotional Awareness’ (*F*(1, 62) = 7.88, *p* < .01, *η²* = .11, power = 0.79), and ‘Self-Regulation’ (*F*(1, 62) = 11.55, *p* < .01, *η*² = .16, power = 0.92). ‘Body Listening’ showed a marginal group difference (*F*(1, 62) = 3.97, *p* = .051, *η²* = .06, power = 0.50), while ‘Not-Distracting,’ ‘Not-Worrying,’ and ‘Trusting’ were not significant (all *p* > .1 for relevant comparisons). Additionally, MAAS results revealed that the BMAA group had significantly higher levels of mindful awareness compared with the control group (*F*(1, 62) = 7.47, *p* < .01, *η*² = .11, power = 0.77). Overall, these findings indicate that participants in the BMAA group reported greater body awareness and mindfulness than those in the control group (see Table 1).

**Table 1.** Adjusted posttest means, standard deviations, and ANCOVA results for the BMAA and control groups.

	Before BMAA	Control	After BMAA	Control	ANCOVA *F* value
Male (*n*)	8	14			
Female (*n*)	30	13			
Age	21.61 (3.07)	18.89 (1.91)			
**Body awareness**					
BAAI	92.74 (11.96)	95.74 (12.76)	99.87 (11.16)	94.85 (13.99)	7.45**
MAIA					
Noticing	3.64 (0.69)	3.40 (0.91)	3.90 (0.50)	3.47 (0.95)	5.35*
Not-distracting	1.59 (0.51)	1.97 (0.65)	1.74 (0.63)	1.97 (0.54)	0.15
Not-worrying	1.85 (0.80)	1.70 (1.06)	1.95 (0.81)	1.67 (1.09)	0.92
Attention regulation	3.10 (0.59)	3.15 (0.73)	3.59 (0.55)	3.20 (0.89)	4.70*
Emotional awareness	3.72 (0.54)	3.55 (0.77)	4.02 (0.60)	3.47 (0.94)	7.88**
Self-regulation	3.17 (0.71)	3.03 (1.15)	3.86 (0.66)	3.06 (1.23)	11.55**
Body listening	3.17 (0.85)	3.40 (0.94)	3.84 (0.67)	3.39 (1.10)	3.97 ms.
Trusting	3.76 (0.78)	3.78 (0.99)	4.18 (0.63)	3.85 (0.99)	2.62
Mindful awareness					
MAAS	65.66 (11.88)	66.89 (8.67)	69.00 (9.35)	64.63 (11.34)	7.47**
**Positive embodiment**					
**EES**					
Total	121.47 (18.79)	116.30 (14.64)	126.11 (14.74)	115.22 (17.93)	5.57*
AF	23.66 (5.00)	25.67 (4.84)	25.55 (4.81)	24.33 (5.92)	7.12*
EESD	13.05 (2.24)	13.56 (2.75)	14.00 (1.68)	13.19 (2.60)	7.60**
ASC	23.08 (3.08)	20.15 (1.51)	24.13 (3.01)	19.74 (1.79)	21.17***
BUA	26.61 (4.72)	25.19 (4.39)	28.58 (4.39)	25.19 (6.09)	5.41*
PBCC	21.92 (2.71)	22.26 (4.28)	22.97 (3.40)	22.96 (4.49)	0.08
RO	11.05 (1.64)	9.48 (1.55)	10.87 (1.53)	9.81 (1.52)	2.28
**Mental health**					
AMHS	94.08 (18.42)	101.70 (13.59)	101.00 (16.32)	99.44 (16.55)	3.83 ms.
TAS20	47.32 (8.60)	51.44 (8.89)	43.13 (9.59)	50.81 (11.13)	5.10*
STAI-T	48.26 (10.58)	43.74 (8.47)	44.76 (10.02)	43.67 (10.96)	2.09
SWLS	23.82 (4.68)	25.37 (5.71)	25.26 (4.28)	25.26 (6.37)	1.21

This table presents the results of ANCOVA comparing post-intervention outcomes between the BMAA and control groups, with pretest scores entered as covariates. Measures include the Body Awareness Ability Inventory (BAAI); Multidimensional Assessment of Interoceptive Awareness (MAIA); Mindful Attention Awareness Scale (MAAS); Experience of Embodiment Scale (EES) and its subscales—Agency and Functionality (AF), Experience and Expression of Sexual Desire (EESD), Attuned Self-Care (ASC), Body Unencumbered Adjustment (BUA), Positive Body Connection and Comfort (PBCC), and Resisting Objectification (RO); Adult Mental Health Scale (AMHS); Toronto Alexithymia Scale–20 (TAS-20); State–Trait Anxiety Inventory–Trait (STAI-T); and Satisfaction with Life Scale (SWLS). Statistical significance for ANCOVA results is denoted as *p < .05, **p < .01, ***p < .001. Marginal significance is indicated as ms. (*p* = .055).

**Within-experimental group change.** Paired-sample analyses further examined pre–post changes within the experimental group following BMAA training. Results showed a significant increase in overall body awareness as measured by the BAAI (*t*(37) = −3.78, *p* < .01, Cohen’s *d* = 0.62). Consistently, MAAS scores indicated a significant improvement in mindful awareness after the intervention (*t*(37) = −2.40, *p* < .05, Cohen’s *d* = 0.31).

For MAIA subscales, repeated-measures MANOVA demonstrated notable enhancements across multiple dimensions. Significant improvements were observed in ‘Noticing’ (*F*(1, 37) = 4.34, *p* < .05, *η*² = .11), ‘Attention Regulation’ (*F*(1, 37) = 25.61, *p* < .001, *η*² = .41), ‘Emotional Awareness’ (*F*(1, 37) = 8.55, *p* < .01, *η*² = .19), ‘Self-Regulation’ (*F*(1, 37) = 38.83, *p* < .001, *η*² = .51), ‘Body Listening’ (*F*(1, 37) = 21.42, *p* < .001, *η*² = .37), and ‘Trusting’ (*F*(1, 37) = 10.90, *p* < .01, *η*² = .23). In contrast, ‘Not-Distracting’ and ‘Not-Worrying’ did not reach statistical significance (*F*(1, 37) = 3.16, *p* > .05; *F*(1, 37) = 0.43, *p* > .1, respectively).

Collectively, the converging evidence from both between-group and within-experimental group analyses demonstrates that BMAA training consistently elevates body awareness and mindfulness, particularly in attentional, emotional, and self-regulatory dimensions of interoceptive awareness. (see Table 1).

This table presents the results of ANCOVA comparing post-intervention outcomes between the BMAA and control groups, with pretest scores entered as covariates. Measures include the Body Awareness Ability Inventory (BAAI); Multidimensional Assessment of Interoceptive Awareness (MAIA); Mindful Attention Awareness Scale (MAAS); Experience of Embodiment Scale (EES) and its subscales—Agency and Functionality (AF), Experience and Expression of Sexual Desire (EESD), Attuned Self-Care (ASC), Body Unencumbered Adjustment (BUA), Positive Body Connection and Comfort (PBCC), and Resisting Objectification (RO); Adult Mental Health Scale (AMHS); Toronto Alexithymia Scale–20 (TAS-20); State–Trait Anxiety Inventory–Trait (STAI-T); and Satisfaction with Life Scale (SWLS). Statistical significance for ANCOVA results is denoted as *p < .05, **p < .01, ***p < .001. Marginal significance is indicated as ms. (*p* = .055).

### Positive embodiment

**Between-group comparison.** ANCOVA results for the EES total score indicated a significant group effect, with the BMAA group showing higher adjusted post-intervention scores than the control group (*F*(1, 62) = 5.57, *p* < .05, *η²* = .08, power = 0.64). Among the six subscales, significant group differences were observed in AF (*F*(1, 62) = 7.12, *p* < .05, *η²* = .10, power = 0.74), EESD (*F*(1, 62) = 7.60, *p* < .01, *η²* = .11, power = 0.77), ASC (*F*(1, 62) = 21.17, *p* < .001, *η²* = .26, power = 0.99), and BUA (*F*(1, 62) = 5.41, *p* < .05, *η²* = .08, power = 0.63), while PBCC and RO showed no significant group differences (both *p* > 0.1).

**Within-experimental group change.** Paired-sample analysis of the EES total score showed no significant pre–post change within the experimental group (*t*(37) = −1.43, *p* > .1). However, repeated measures MANOVA revealed significant improvements in several EES subscales following BMAA training. Specifically, significant increases were found in AF (*F*(1, 37) = 8.48, *p* < .01, *η*² = .19), EESD (*F*(1, 37) = 8.89, *p* < .01, *η*² = .19), and BUA (*F*(1, 37) = 7.27, *p* < .05, *η*² = .16). The ASC subscale showed a marginal effect (*F*(1, 37) = 3.90, *p* = .056, *η*² = .10), while PBCC and RO remained non-significant (*F*(1, 37) = 3.76, *p* = .06; *F*(1, 37) = 0.39, *p* > .1, respectively).

Overall, the converging evidence from both between-group and within-experimental group analyses clearly demonstrates that BMAA training leads to substantially higher levels of embodiment in AF, EESD, and BUA, with ASC showing a marginal increase, whereas PBCC and RO remained unchanged.

### Mental health

**Between-group comparison.** ANCOVA analyses were conducted to examine group differences in mental health–related measures. For the AMHS, the adjusted post-intervention scores were marginally higher in the BMAA group compared with the control group (*F*(1, 62) = 3.83, *p* = .055, *η*² = .06, power = 0.49), suggesting a potential mental health benefit associated with BMAA participation. A significant group difference was also found for the TAS-20, with the BMAA group showing lower adjusted scores, indicating better emotional differentiation and expression relative to the control group (*F*(1, 62) = 5.10, *p* < .05, *η*² = .08, power = 0.60). No significant group differences were observed for the STAI-T or SWLS (*p* > .1 for relevant comparisons).

**Within-experimental group change.** Paired-sample t-tests were conducted to examine pre–post changes within the BMAA group. Results showed a significant increase in mental health as measured by the AMHS (*t*(37) = −2.61, *p* < .05, Cohen’s *d* = 0.40). Consistently, alexithymia levels assessed by the TAS-20 significantly decreased following training (*t*(37) = 3.38, *p* < .01, Cohen’s *d* = 0.46), indicating improved emotional differentiation and expression. Moreover, participants demonstrated significantly lower trait anxiety post-training on the STAI-T (*t*(37) = 2.80, *p* < .01, Cohen’s *d* = 0.34), alongside a significant increase in life satisfaction as reflected by SWLS scores (*t*(37) = −2.07, *p* < .05, Cohen’s *d* = 0.32).

**Figure 2.**
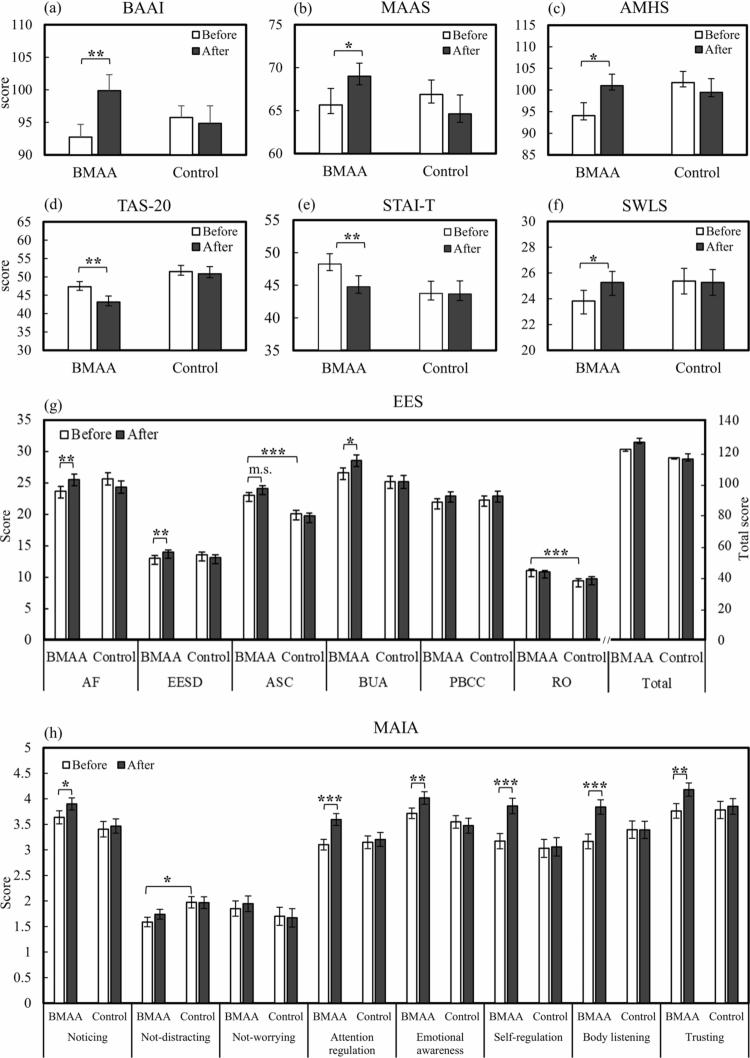
Pre–post changes in psychological outcomes for the BMAA and control groups. Pretest and posttest scores for the BMAA and control groups across all outcome measures. Although both groups are displayed for visual comparison, the significance markers (*, **, ***) indicate within-group pre–post changes in the BMAA group only, based on paired-samples t-tests and repeated-measures MANOVA. Panels (a)–(f) show outcomes for the Body Awareness Ability Inventory (BAAI), Mindful Attention Awareness Scale (MAAS), Adult Mental Health Scale (AMHS), Toronto Alexithymia Scale–20 (TAS-20), State–Trait Anxiety Inventory–Trait (STAI-T), and Satisfaction with Life Scale (SWLS). Panel (g) displays the Experience of Embodiment Scale (EES) and its subscales: Agency and Functionality (AF), Experience and Expression of Sexual Desire (EESD), Attuned Self-Care (ASC), Body Unencumbered Adjustment (BUA), Positive Body Connection and Comfort (PBCC), and Resisting Objectification (RO). Panel (h) presents the eight subscales of the Multidimensional Assessment of Interoceptive Awareness (MAIA). Error bars represent standard deviations. Significance levels: *p < .05, **p < .01, ***p < .001; marginal significance noted as m.s. (*p* = .055).

As a whole, the consistency between the between-group and within-experimental group findings demonstrates that BMAA training is associated with better mental health, reduced alexithymia, lower anxiety, and higher life satisfaction, underscoring its beneficial impact on psychological well-being (see Figure 2).

## Discussion

This study investigated the effects of BMAA training on healthcare students’ body awareness, mindfulness, embodiment, and mental health using eight validated measures. Between-group analyses showed that students who participated in BMAA reported higher post-intervention levels of body and mindful awareness, positive embodiment, and mental health compared with controls. Within-group analyses further revealed significant pre–post gains in the same domains, reflecting consistent improvements in body–mind integration and psychological well-being. Overall, these findings suggest that BMAA may serve as an effective embodied approach to promote holistic health and resilience among healthcare students.

### Effects of BMAA training on body awareness and mindfulness

The improvements observed across body-awareness measures indicate that BMAA strengthens interoceptive sensitivity and bodily attunement. This pattern aligns well with the method’s emphasis on axial alignment, mindful movement, and breath regulation. BAAI results showed clearer differentiation between tension and relaxation and greater awareness of bodily discomfort, suggesting improved discrimination of internal bodily states. Consistent with this, multiple MAIA dimensions—including Noticing, Attention Regulation, Emotional Awareness, Self-Regulation, Body Listening, and Trusting—demonstrated significant gains, reflecting an enhanced capacity to monitor and interpret interoceptive signals. In contrast, Not-Distracting and Not-Worrying did not exhibit significant changes. As these subscales capture cognitive attitudes toward discomfort, their slower improvement may indicate that such attitudes require longer-term practice or more explicit cognitive strategies. Their upward trends nevertheless suggest that extended BMAA engagement may gradually foster a more accepting and less reactive stance toward bodily sensations.

Taken together, the improvements in interoceptive and self-regulatory capacities form a coherent basis for understanding the observed changes in mindful awareness. Beyond these gains in body awareness, participants also demonstrated higher levels of mindfulness after training, with MAAS scores indicating an enhanced ability to sustain attention to present-moment experience. This increase in mindfulness is consistent with the attentional redirection and interoceptive monitoring repeatedly practiced during BMAA sessions.

The continual engagement with bodily cues inherent in BMAA may therefore cultivate a more stable and integrated form of awareness that spans both interoceptive and attentional domains. This overall pattern aligns with prior research on body-awareness therapies (Gyllensten et al., 2003; Mehling et al., 2011) and mindfulness-based interventions (Flink et al., 2009; Goyal et al., 2014; Kabat-Zinn, 2003), which consistently associate enhanced interoception with reduced negative affect, greater vitality, and improved psychological well-being (Farb et al., 2015; Grossman et al., 2004).

### Enhancement of positive embodiment through BMAA

The pattern of improvements across the EES total score and the AF, EESD, ASC, and BUA subscales suggests that BMAA fosters a more empowered and adaptive sense of embodiment. These changes are also consistent with BMAA’s emphasis, which encourage students to engage with their bodies in a more coordinated and empowered manner. Rather than simply increasing sensory awareness, these movement-based elements promote a felt sense of bodily agency, functional confidence, and fluidity in everyday actions. Participants also appeared to cultivate more responsive and compassionate self-care practices—such as recognising cues of hunger, rest, or fatigue and responding appropriately—which aligns with prior research on attuned self-care as a core component of positive embodiment (Burychka et al., 2021; Piran et al., 2020; Piran, 2017). This trajectory echoes earlier findings that embodied awareness supports a balanced and flexible relationship with one’s body, promoting vitality, functional engagement, and emotional grounding (Alleva & Tylka, 2021; Gyllensten et al., 2010). By reinforcing a sense of presence within the body, BMAA may help participants feel more connected to their physical capacities and internal signals, thereby enhancing psychological stability and resilience.

In contrast, PBCC and RO did not show notable changes. The limited effects on PBCC suggest that developing a stable sense of bodily comfort may require longer practice or more cognitively oriented strategies, as comfort often reflects deeper patterns of self-evaluation and habitual reactivity. Similarly, resistance to objectification (RO) may be less amenable to short-term embodied practices, given its roots in sociocultural pressures and internalised appearance norms (Fredrickson & Roberts, 1997; Tiggemann & Lynch, 2001). Complementary interventions—such as reflective dialogue, psychoeducation, or cognitive reframing—may be necessary to meaningfully shift these more entrenched dimensions of embodiment (Alleva et al., 2019).

Overall, the findings indicate that BMAA can facilitate the development of positive embodiment, which has been linked to lower anxiety and depression and greater psychological resilience (Piran, 2016; Tylka & Wood-Barcalow, 2015). Through sustained engagement with bodily sensations and mindful self-care, participants may strengthen emotional regulation, self-esteem, and a sense of internal coherence—qualities that are particularly crucial for healthcare students navigating high academic and emotional demands (Hojat et al., 2011; Neumann et al., 2011; Sanjaya et al., 2024). By fostering deeper bodily connection and acceptance, BMAA may also promote a more grounded and integrated sense of identity (Dolezal, 2015; Rosendahl et al., 2021; Tylka & Piran, 2019).

### Impact of BMAA training on mental health

The pattern of improvements across multiple mental health indicators suggests that BMAA may support reduced anxiety vulnerability, emotional clarity, and overall psychological well-being in healthcare students. Participants who engaged in BMAA showed signs of enhanced emotional differentiation and expression, reflected in better overall mental health and reduced alexithymia—capacities that are closely tied to interoceptive clarity and embodied emotional awareness (Mano et al., 2019; Stoppard & Gunn Gruchy, 1993). BMAA’s emphasis on sustained attention to bodily sensations, regulated breathing, and grounded posture may help individuals recognise emerging emotional cues more accurately and respond in a less reactive, more adaptive manner.

These patterns further align with evidence from other body-centred practices—such as yoga, tai chi, and mindfulness-based training—that highlight the role of embodied awareness in stress reduction and affect regulation (Farb et al., 2013; Khalsa & Butzer, 2016). Unlike conventional mindfulness programmes such as Mindfulness-Based Stress Reduction (MBSR) (Irving et al., 2009; Kabat-Zinn, 2003), BMAA cultivates mindful awareness through gentle, continuous movement rather than seated meditation. This dynamic, movement-based format may be particularly supportive for individuals who experience physical tension, restlessness, or difficulty maintaining attention in static practices (Lien et al., 2019; Yeh et al., 2023). By grounding attention in axial alignment and rhythmic body–breath coordination, BMAA may deepen interoceptive sensitivity and facilitate down-regulation of physiological arousal.

Moreover, the learning environment fostered in BMAA—characterised by non-judgement, internal focus, and the deliberate absence of mirrors or appearance-related cues—encourages participants to shift attention from external evaluation to internal sensation. This inward orientation may strengthen bodily confidence, self-compassion, and emotional regulation, skills essential for healthcare students navigating high academic pressure and emotionally demanding clinical responsibilities (Auerbach et al., 2016; Eisenberg et al., 2009).

Taken together, these findings indicate that BMAA may function as a promising embodied approach for enhancing psychological resilience and self-regulation in healthcare education. By cultivating interoceptive clarity, emotional awareness, and grounded presence, BMAA offers a movement-based pathway to support mental health in populations vulnerable to stress and burnout.

### Relevance to clinical contexts

Healthcare education unfolds within emotionally intense, high-stakes environments, where developing resilience and emotion-regulation capabilities during training correlates with enhanced professional performance (Sanjaya et al., 2024). Consistent with this, physician empathy has been empirically linked with better patient outcomes and higher satisfaction—underlining the clinical importance of emotional attunement and effective communication (Hojat et al., 2011; Neumann et al., 2011). Intervention research further supports this connection: randomised controlled trials and longitudinal programmes demonstrate that mindfulness- and awareness-based trainings reduce distress and burnout and can bolster empathy and patient-centred communication among health-care trainees (De Vibe et al., 2013; Krasner et al., 2009). Recent systematic reviews also suggest that resilience and stress-management programmes yield small-to-moderate improvements in stress, anxiety, and wellness in medical learners (Angelopoulou & Panagopoulou, 2022; Bennett-Weston et al., 2024; Kaisti et al., 2024).

Within this context, Body‑Mind Axial Awareness (BMAA)—by explicitly engaging body awareness and interoceptive attention—may provide a movement-based pathway to similar outcomes (e.g. reduced anxiety and enhanced emotional processing observed in our study), thereby complementing existing classroom-based resilience and mindfulness curricula designed for healthcare students.

### Limitations and future directions

This study presents encouraging findings, yet several methodological limitations should be noted. First, the lack of random assignment limits internal validity. The experimental group consisted of students who voluntarily enroled in an elective course, whereas the control group was recruited through convenience sampling. Although ANCOVA adjusted for baseline differences, unmeasured characteristics—such as motivation, emotional openness, or prior interest in body–mind practices—may still have contributed to group differences. Second, the groups were not demographically matched. The BMAA group was older and had a substantially higher proportion of female participants. Such imbalances are important because prior research shows that women generally demonstrate higher interoceptive sensitivity and greater attention to internal bodily states (Grabauskaitė et al., 2017; Longarzo et al., 2021; Murphy et al., 2019). The overrepresentation of women in the BMAA group may therefore have partially amplified improvements in interoception-related outcomes. Future studies should use randomised controlled designs or matched sampling to reduce demographic confounding. Third, the study relied exclusively on self-report questionnaires, which are vulnerable to response biases. Incorporating behavioural or physiological indicators—such as heart rate variability or interoceptive accuracy tasks—would provide more objective evidence of BMAA’s effects. Finally, without long-term follow-up, the durability of the observed improvements remains unclear; future longitudinal work should examine whether benefits are sustained over time.

## Conclusion

This study demonstrates that BMAA training is associated with improvements in body awareness, mindfulness, positive embodiment, and psychological functioning among healthcare students. These findings contribute to the growing evidence that embodied, movement-based practices can play a meaningful role in supporting students’ emotional regulation and well-being. While the results highlight the promise of BMAA as an educational intervention within healthcare training, the conclusions should be interpreted within the context of the study sample. Even so, the present findings underscore the potential value of incorporating holistic, body-centred approaches such as BMAA into healthcare curricula to promote student well-being and resilience.

## Data Availability

The data used in this study are available from the corresponding author upon reasonable request.
